# The effects of mother-infant bonding on children's strengths and difficulties

**DOI:** 10.1016/j.heliyon.2025.e41727

**Published:** 2025-01-06

**Authors:** Tomoko Kawasaki, Yoshikazu Noda, Yoshiyuki Hirano, Akiko Kawanami, Kenichi Sakurai, Chisato Mori, Eiji Shimizu

**Affiliations:** a Research Center for Child Mental Development, Chiba University, 1-8-1, Inohana, Chuo-ku, Chiba, 260-0856, Japan; b United Graduate School of Child Development, Osaka University, Kanazawa University, Hamamatsu University School of Medicine, Chiba University and University of Fukui, 1-8-1, Inohana, Chuo-ku, Chiba, 260-0856, Japan; c Center for Preventive Medical Sciences, Chiba University, 1-33, Yayoicho, Inage-ku, Chiba, 263-8522, Japan; d Graduate School of Medicine, Chiba University, 1-8-1, Inohana, Chuo-ku, Chiba, 260-0856, Japan

**Keywords:** Latent class analysis, Mother-infant bonding, Postpartum depression, Strengths and difficulties questionnaire, Birth cohort

## Abstract

**Background:**

Several studies have shown that parental relationships between infants and their caregivers, and their mental health condition in childhood, influence children's development. The present study aimed to predict the effects of maternal mental health and mother-infant bonding at 10 months of age on Japanese children's behavioral development at 5 years. We analyzed factors including maternal mental health and mother-infant bonding to predict subtype classes as a prospective cohort study.

**Methods:**

Data from Japan's Chiba Study of Mother and Child Health (C-MACH) were used. The Japanese versions of the Mother-to-Infant Bonding Scale (MIBS) and Strengths and Difficulties Questionnaire (SDQ) were administered to 275 consenting mothers and their children. We used latent class analysis (LCA) to classify the children's characteristics. We analyzed the relationship between sociodemographics, maternal mental states, and latent classes using ANOVA and multinomial logistic regression.

**Results:**

Three latent classes were found: “asocial” (41.3 %), “well-adjusted” (20.3 %), and “highly difficult” (38.4 %). Children in the “asocial” class had a low probability for all behaviors (emotional, conduct, hyperactivity, peer problems, and prosocial behaviors). Children in the “highly difficult” class were more likely to exhibit conduct problems, hyperactivity, and peer problems as well as fewer prosocial behaviors. In addition, there were more males in the “asocial” class than in the “well-adjusted” class (Odds Ratio (OR) = 0.30, 95 % confidence interval (CI) [0.11; 0.83]), and they had higher MIBS 10-month scores (OR = 1.39, 95%CI [1.00; 1.94]).There were fewer females in the “highly difficult” than in the “well-adjusted” class (OR = 0.16, 95 % CI [0.06; 0.43]), and they had higher MIBS 10-month scores (OR = 1.65, 95%CI [1.21; 2.26]).

**Conclusions:**

The results suggest that insufficient mother-infant bonding at 10 months may contribute to males’ asocial behavior at five years. These findings can help parents better understand and manage these issues in their children for prevention and intervention.

## Highlights

• Subtypes of strengths and difficulties were explored in Japanese children.
• Two of the main classifications identified were “asocial” and “highly difficult”.
• These two classes accounted for approximately 80 % of the children studied.
• Exhibiting impaired maternal bonding and being a male predict these two classes.
• Strong mother-infant bonding and being a female predict prosocial behaviors.

## 1 Introduction

The prevalence of mental disorders among children aged 1–7 years is reported to be 20.1 %, with the most common disorders being oppositional defiant disorder and attention deficit hyperactivity disorder [1]. This has serious implications for children's future mental health [2], as early intervention is critical to treating mental health disorders [3]. In particular, it has been studied that early detection and appropriate support for autism spectrum disorder (ASD) and attention deficit hyperactivity disorder (ADHD), which often show characteristic behaviors and social difficulties, can promote later development among children aged 5 years [4]. For this reason, in recent years, the Japanese government has been recommending health checks for 5-year-olds to evaluate their emotional and behavioral development. Identifying early signs of these disorders is crucial and can be facilitated using tools such as the Strengths and Difficulties Questionnaire (SDQ) created by Goodman [5]. The SDQ quantifies problem behaviors by focusing on children's positive and negative attitudes, offering an effective screening tool for parents and healthcare providers. Additionally, the SDQ has been shown to be a reliable tool for assessing the mental health of individuals in a variety of settings, providing clear correlations with specific mental health conditions [6]. SDQ in preschool age can identify children with a highly increased risk of being diagnosed and/or treated for neurodevelopmental disorders such as ADHD at school age [7]. In Japan, it is widely used to diagnose developmental disorders, psychological conditions, and mental disorders in children. Matsuishi [8] reported that Japanese males score significantly higher in terms of the total difficulties score (TDS) of the SDQ as well as the mean scores for conduct problems, hyperactivity, and peer problems. In contrast, females score significantly higher on emotional and prosocial aspects. Meanwhile, another study reported that there were no significant differences between males and females in any of the categorical items [9]. Therefore, the effectiveness of the SDQ regarding analyzing gender differences is debatable. Additionally, birth order affects children's mental health, with firstborns having higher “conduct problem” scores [10].

Relationships between infants and their parents or caregivers play an essential role in their social and emotional development [11]. Children with secure relationships with their parents or other primary caregivers have fewer internalizing and externalizing behaviors [12], are more socially competent, and have better-quality friendships [13]. Moreover, children's healthy development is compromised when their parents' mental health deteriorates [14]. Children with mothers who have depression are more likely to experience internalizing and externalizing problems [15]. For example, in Japan, postpartum depression (PPD) that persists in infancy is significantly associated with children's internalization problems at age six [16]. Fuchs et al. [17] reported that bonding failure at 14 months predicted difficulties reflected on the SDQ at the age of five. However, Fuchs dealt only with the four subscales of child difficulties. As the present analysis takes children's strengths into account, we complemented Fuchs's method and examined how maternal bonding affects children's strengths and weaknesses. Furthermore, because no studies have examined the relationship between the Edinburgh Postnatal Depression Scale (EPDS) or the Mother-to-Infant Bonding Scale (MIBS) and the SDQ, we assumed that these two factors could be predictors in our analysis.

Recently, latent class analysis (LCA) as a person-centered approach has been used for subtyping classes with similar SDQ tendencies [[18], [19], [20]]. Using LCA, we can understand the characteristic patterns of children on different SDQ subscales. In a survey of children aged 7–12 years in Spain, four subscales, excluding the prosocial scale, were used in LCA to identify five classes of SDQ: “high difficulties,” “introverted symptoms,” “extraverted symptoms,” “hyperactivity,” and “adaptive” [19]. In China, LCA of SDQ scores on five subscales, including the prosocial scale, was conducted on adolescents aged 11–18 years. They were divided into three classes: highly difficult, uncooperative, and well-adjusted [18]. Using LCA with four subscales, excluding the prosocial scale, a survey of families with children aged 6–11 years in Japan showed three classes, a “risk group,” “moderate group,” and “normal group,” and that family activities were significantly related to the normal group [20].

**Fig. 1:**
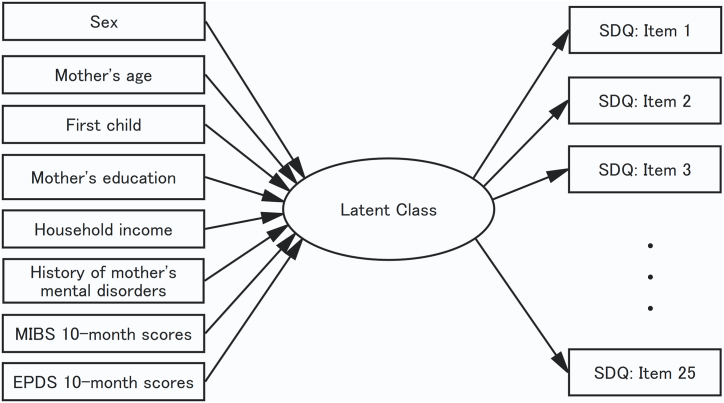
Latent class model of the current study. The SDQ scores were predicted by social demographic characteristics and maternal psychological measurement. Note. MIBS, Mother-to-Infant Bonding Scale; EPDS, Edinburgh Postnatal Depression Scale; SDQ, The Strengths and Difficulties Questionnaire.

This study aimed to determine whether there were significant populations with similar emotional and behavioral problems and strengths among Japanese 5-year-olds using LCA. In addition, factors (household income, educational background, maternal age, child's sex, whether the child was firstborn, EPDS score, and MIBS score) that influenced latent classes were examined. We hypothesized that classes with characteristics different from those of the SDQ subscales exist. We expected EPDS and MIBS scores to be significant predictors of class membership (Fig. 1).

## 2 Methods

### 2.1 Participants

This study used data from a birth cohort study, the Chiba Study of Mother and Child Health (C-MACH) in Japan [21]. The study population included pregnant women, their marital partners, and their children. Participants were recruited prior to 13 weeks of gestation at three hospitals in Chiba and Saitama Prefectures in Japan from February 2014 to June 2015.

**Fig. 2:**
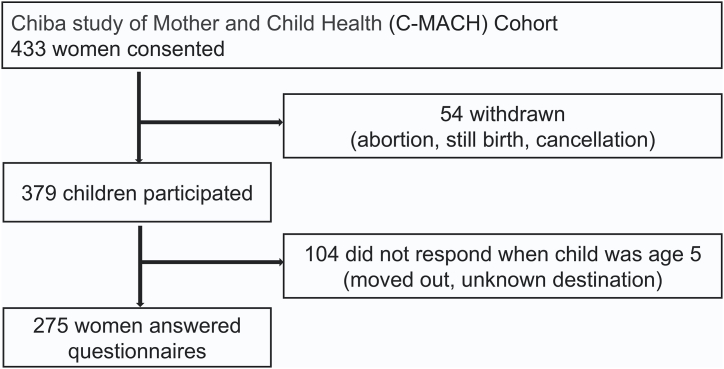
Flowchart of participants who met inclusion/exclusion criteria.

Self-reported questionnaires were collected from participants at various time points before and after childbirth. Four hundred and thirty-three mothers participated in the main cohort study, and data from 379 mother-child pairs were available at birth. For the analysis, we included 275 pairs who responded to the MIBS and EPDS when their children reached the age of 10 months and the SDQ when the child reached 5-year-olds (Fig. 2).

### 2.2 Measurements

#### 2.2.1 Children's Strengths and Difficulties Questionnaire

The SDQ is a brief self-report screening instrument to measure children's psychological adjustment. Developed by Goodman [5](supplementary file 1) and translated into Japanese [8], the SDQ comprises 25 items grouped into five subscales: emotional symptoms, conduct problems, hyperactivity, peer problems, and prosocial behavior. Items are rated on a three-point scale ranging from 0 (not true) to 2 (certainly true). Higher prosocial behavior subscale scores on the SDQ indicate greater strength. The Japanese version of the SDQ for 4-to-12-year-olds showed that the Cronbach's alpha for items in the parent assessment was 0.61 for emotional problems, 0.52 for conduct problems, 0.75 for hyperactivity, 0.52 for peer problems, and 0.69 for strengths in prosocial behavior [8]. In the present study, Cronbach's alpha of 5-year-olds was as follows: α = .67 for emotional problems, α = .55 for behavioral problems, α = .74 for hyperactivity, α = .56 for peer problems, and α = .70 for prosocial behavior.

#### 2.2.2 The Japanese version of mother-to-Infant Bonding Scale (MIBS)

The MIBS comprises 10 items investigating mothers' emotional bonding with children [22,23] (supplementary file 2). Self-reported questions are rated on a four-point scale, from 0 = not at all to 3 = very much, with scores ranging from 0 to 30. Higher scores indicate reduced or absent emotional bonding. In Japanese studies, the MIBS scale has been used from the period immediately after childbirth to 12 months after childbirth [24]. In Fuchs's study, the MIBS score at 14 months was analyzed as a predictor of the SDQ [17]. Based on these two studies, we chose the measurement of maternal mental health (MIBS, EPDS) data at 10 months after childbirth, which is the closest time point we have. According to Tokuda et al. [24], Cronbach's alpha for the MIBS was 0.73 at 12 months, compared to 0.78 at 10 months in the current study.

#### 2.2.3 Edinburgh Postnatal depression scale (EPDS)

The EPDS comprises 10 items and was developed by British psychiatrist John Cox to screen for postpartum depression [25](Supplementary file 3). Self-reported questions are rated on a four-point scale: 0 = not at all to 3 = all the time. Scores range from 0 to 30. The Japanese version of the EPDS [26] has been widely used in several studies as a screening for perinatal depression in Japanese women [[24], [25], [26]]. In Japan, Cronbach's alpha for EPDS was 0.74 [26] at three months postpartum, whereas in the present study, it was 0.81 at 10 months postpartum. We used maternal mental health EPDS and MIBS scores at 10 months postpartum as potential confounders for the SDQ.

#### 2.2.4 Covariates

We analyzed the relationship between the scores and the following variables: sex of the child (1 = male, 2 = female) household income (less than 2 million yen, 2–4 million yen, 4–6 million yen, 6–8 million yen, 8–10 million yen, 10–12 million yen, 12–15 million yen, less than 20 million yen, 20 million yen or more, no response), mother's age, educational background (last education was junior high school, high school, vocational school/junior college/technical college, university/graduate school or higher, no response), history of mental illness (depression, autonomic dysreflexia, or anxiety; 0 = no history, 1 = have history), and siblings (1 = first child, 2 = not first child).

### 2.3 Statistical analysis

Based on LCA, we estimated the number of classes the SDQ pattern could be divided into. LCA is a person-centered analysis that uses binary indicators to identify latent response patterns and assign individuals to specific classes based on those patterns. Multinomial logistic regression in R3STEP was performed to assess the association between latent classes and sex, siblings, mother's age, mother's education, household income, MIBS, and EPDS [27]. All analyses were based on a p-value less than 0.05. We applied the full information maximum likelihood (FIML) method to deal with missing values, assuming that the data are missing completely at random (MCAR) or missing at random and analyses can be performed based on the available data without missing values. Therefore, if the data were not missing at random (NMAR), the results might have been misinterpreted. Analyses to determine the distribution of the data, the MCAR test, chi-square test, ANOVA, and effect size calculations were conducted using Stata 17 (Stata Corp LP, College Station, TX, USA). The LCA and multinomial logistic regression were conducted using Mplus 8 (Muthén & Muthén, Los Angeles, CA, USA).

## 3 Results

### 3.1 Latent class model Fitting

Little's Missing Completely at Random (MCAR) test was applied to the dataset and yielded a non-statistically significant result (χ^2^ = 375.22, p = .37), indicating that missing data within the dataset can be assumed to not be completely random. Statistical criteria and class interpretability were considered to determine the most suitable model, and BIC, LMR, and BLRT exhibited three-class solutions. The entropy was greater than 0.80, and the classes were theoretically distinct. Therefore, the three-class solution was the best fit for the data (Table 1).

**Table 1:** Model fit criteria for latent class models of psychopathological symptoms measured by the Strengths and Difficulties Questionnaire in a sample of Japanese 5-year-old children (N = 275).

	1 class	2 classes	3 classes	4 classes	5 classes
Free parameters	25	51	77	103	129
AIC	7076.323	6634.286	6528.022	6460.425	6427.798
BIC	7166.743	6818.741	**6806.514**	6832.953	6894.362
SSABIC	7087.472	6657.030	6562.362	6506.360	6485.327
VLMR, *p* value		0.000	**0.019**	0.270	0.330
BLRT, *p* value		0.000	0.000	0.000	0.000
Entropy		**0.856**	**0.844**	**0.863**	**0.876**

Note. AIC, Akaike Information Criterion; BIC, Bayesian Information Criterion; SSABIC, Sample-Size-Adjusted Bayes Information Criterion; VLMR, Vuong-Lo-Mendell-Rubin Likelihood Ratio Test; BLRT, Bootstrapped Likelihood Ratio Test.

### 3.2 Class assignments

**Fig. 3:**
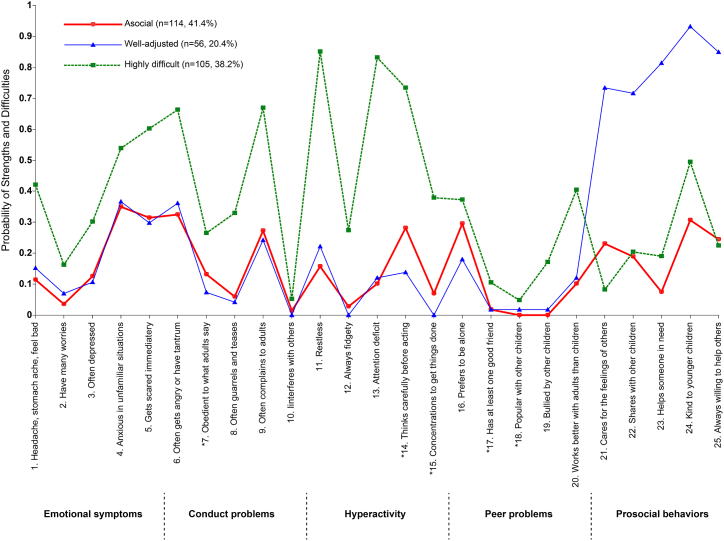
Estimated probabilities of four latent classes' strengths and difficulties in five-year old Japanese children.

The first class (asocial; n = 114) had a low probability of exhibiting all behaviors (emotional, conduct, hyperactivity, peer problems, and prosocial behavior). The second class (well-adjusted; n = 56) had the fewest children and was characterized by a low probability of exhibiting the following four behaviors: emotional symptoms, conduct problems, hyperactivity, and peer problems. However, it was also characterized by a high likelihood of exhibiting prosocial behavior. The third class (highly difficult; n = 105) had a moderate probability of emotional symptoms but a substantial probability of exhibiting conduct problems, hyperactivity, and peer problems as well as a low probability of social behavior (Fig. 3).

### 3.3 Association between SDQ subscales and latent classes

The sociodemographic variables and SDQ subscale scores are shown in Table 2, Table 3. Based on the results of the ANOVA, the SDQ subscale scores (no symptoms = 0, some symptoms = 1) were evaluated by the latent class. Latent classes differed significantly according to sex, mother's age, MIBS 10-month scores, and EPDS 10-month scores. There were no significant differences in latent class regarding whether the child was the first child, maternal education, household income, or maternal history of mental illness. MIBS scores at 10 months were highest in the “highly difficult” class (3.47, SD = 3.40) and lowest in the “well-adjusted” (1.29, SD = 1.67) and “asocial” (1.98, SD = 2.17) classes. The scores for “well-adjusted” were significantly lower than those of the other two classes. The effect sizes (Hedge's g) between “highly difficult” and “asocial” or “well-adjusted” were 0.53 and 0.74, respectively. There was a significant difference in EPDS scores at 10 months between the “highly difficult” and “well-adjusted” classes. Across all SDQ subscales, we observed significant variations among the clusters, with effect sizes spanning a broad range from low to high, as measured by Hedge's g (ranging from 0.38 to 2.78).

**Table 2:** Sociodemographic variables depending on latent class membership.

		Total	Asocial	Well-adjusted	Highly difficult	F/χ^2^	Effect size *η*^2^/Cramer's V	Post hoc test	
								Direction	Hedge's g
		n = 275	n = 114	n = 56	n = 105				
Sex									
male (%)		142	57 (50.0)	16 (28.6)	69 (65.7)	20.38∗∗∗	0.27		
female (%)		133	57 (50.0)	40 (71.0)	36 (34.3)				
Mother's age, M (SD) (n = 270)		32.62 (4.57)	33.66 (4.24)	32.54 (4.57)	31.35 (4.70)	6.09∗∗	0.44	1 < 3	0.48
First child (%)	Yes	123 (44.7)	42 (36.8)	28 (50.0)	53 (50.5)	4.90	0.13		
	No	152 (55.3)							
Mother's education, N (%)	Junior high school	4 (1.5)	0 (0)	1 (1.8)	3 (2.9)	9.43	0.13		
	High school	53 (19.6)	20 (18.0)	6 (10.7)	27 (26.2)				
	Junior college	114 (42.2)	50 (45.1)	26 (46.4)	38 (36.9)				
	University/graduate school	99 (36.7)	41 (36.9)	23 (41.1)	35 (34.0)				
	Unclear	5 (1.8)							
Household income, N (%)	<2 million yen	1 (0.4)	0 (0)	0 (0)	1 (0.3)	21.59	0.20		
	2-4 million yen	46 (17.7)	20 (18.4)	9 (17.3)	17 (17.2)				
	4-6 million yen	83 (31.9)	29 (26.6)	17 (32.7)	37 (37.4)				
	6-8 million yen	70 (26.9)	31 (28.4)	15 (28.9)	24 (24.2)				
	8-10 million yen	29 (11.2)	10 (9.2)	5 (9.6)	14 (14.1)				
	10-12 million yen	19 (7.3)	13 (11.9)	2 (3.9)	4 (4.0)				
	12-15 million yen	7 (2.7)	2 (1.8)	4 (7.7)	1 (1.0)				
	15-20 million yen	2 (0.8)	1 (0.9)	0 (0)	1 (1.0)				
	>20 million yen	3 (1.1)	3 (2.75)	0 (0)	0 (0)				
	Unclear	15 (5.5)							
History of mother's mental disorders, N (%)	Yes	20 (8.1)	8 (8.4)	5 (9.1)	7 (7.2)	0.19	0.03		
	No	227 (91.9)	87 (91.6)	50 (90.9)	90 (92.8)				
	Unclear	32 (11.6)							

∗*p* < .05, ∗∗*p* < .01, ∗∗∗*p* < .001.

**Table 3:** MIBS, EPDS, and SDQ scores depending on latent class membership.

		Total		Asocial	Well-adjusted	Highly difficult	F/χ^2^	Effect size *η*^2^/Cramer's V	Post hoc test	
									Direction	Hedge's g
		n = 275		n = 114	n = 56	n = 105				
MIBS 10-month scores, M (SD) (n = 253)		2.42 (2.77)		1.98 (2.17)	1.29 (1.67)	3.47 (3.40)	13.74∗∗∗	0.1	1 < 3	0.53
									2 < 3	0.74
EPDS 10-month scores, M (SD) (n = 257)		3.97 (3.45)		3.17 (2.62)	3.61 (3.27)	5.05 (4.05)	8.74∗∗∗	0.06	1 < 3	0.56
									2 < 3	0.38
SDQ, M (SD) (n = 253)	Emotional symptoms	1.64 (1.81)		1.00 (1.14)	1.20 (1.49)	2.56 (2.14)	26.77∗∗∗	0.16	2 < 3	0.70
									1 < 3	0.97
	Conduct problems	2.26 (1.59)		1.73 (1.13)	1.43 (1.19)	3.29 (1.67)	48.03∗∗∗	0.26	1 < 3	1.10
									2 < 3	1.21
	Hyperactivity	3.43 (2.10)		2.35 (1.08)	1.79 (1.34)	5.47 (1.58)	199.13∗∗∗	0.59	2 < 1	0.48
									1 < 3	2.31
									2 < 3	2.44
	Peer problems	1.60 (1.55)		1.26 (1.05)	0.80 (1.18)	2.39 (1.83)	28.31∗∗∗	0.17	1 < 3	0.76
									2 < 3	0.97
	Prosocial behavior	6.24 (2.04)		5.53 (1.44)	9.07 (0.85)	5.51 (1.72)	131.32∗∗∗	0.49	1 < 2	2.78
									3 < 2	2.39
	Total difficulties	8.92 (4.95)		6.34 (2.32)	5.21 (3.19)	13.70 (3.93)	192.49∗∗∗	0.59	1 < 3	2.30
									2 < 3	2.29

∗*p* < .05, ∗∗*p* < .01, ∗∗∗*p* < .001.

### 3.4 Predicting class

Multinomial logistic regression assessed the association between participants’ classification and sociodemographic variables (Table 4).

**Table 4:** Results of multinomial logistic regression predicting latent class membership.

	Asocial (n = 114)		Highly difficult (n = 105)	
	OR	95 % CI	OR	95 % CI
Sex	**0.30**	0.11; 0.83	**0.16**	0.06; 0.43
Mother's age	1.02	0.90; 1.15	0.96	0.85; 1.07
First child	1.44	0.56; 3.71	0.94	0.36; 2.45
Mother's education	0.80	0.41; 1.56	0.53	0.27; 1.04
Household income	1.21	0.88; 1.65	1.23	0.89; 1.69
History of mother's mental disorders	0.82	0.14; 4.83	0.55	0.09; 3.39
MIBS 10-month scores	**1.39**	1.00; 1.94	**1.65**	1.21; 2.26
EPDS 10-month scores	0.87	0.73; 1.05	0.98	0.84; 1.14

Note. OR = odds ratio; CI = confidence interval. Significant results are printed in bold. The reference is “well-adjusted class” for all factors.

Compared to the “well-adjusted” class, the “highly difficult” class had more males (OR = 0.16, 95 % confidence interval (CI) [0.06–0.43]) and greater MIBS 10-month scores (OR = 1.65 [1.21–2.26]). In addition, compared to the “well-adjusted” class, the “asocial” class had more males (OR = 0.30 [0.11–0.83]) and higher MIBS 10-month scores (OR = 1.39 [1.00–1.94]). The results indicated no significant differences among the latent classes of the other analyzed factors (mother's age, history of mental disorders, household income, maternal educational history, whether the child was the first child, and EPDS score at 10 months).

### 3.5 Possibility of selection bias

Comparing the differences between the completed participants (n = 275) and those who dropped out by the age of 5 (n = 104), there were no significant differences in MIBS 10-month or EPDS 10-month scores by the Wilcoxon rank-sum (Mann-Whitney) test between the two groups. Furthermore, using the *t*-test, there was no statistical difference in the age of the mothers between the two groups; the chi-square test showed that there was no statistical difference in the sex of the children, history of mothers' mental disorders, or household income. However, there was a significant difference in the mothers’ education (p = .032) (Supplementary Table 1).

## 4 Discussion

Through LCA, we identified three distinct subtypes from the SDQ responses. To our knowledge, this is the first longitudinal study in Japan that applies LCA to all 25 items of the SDQ within a general population context. In addition, it investigates risk factors associated with patterns of SDQ. A recent Japanese study divided the SDQ into three classes with 20 items, excluding prosocial behavior, and examined the association with family activities [20]. Therefore, the present study is even more comprehensive in its results as it is concerned with SDQ subtypes, including prosocial behavior in 5-year-olds. In this analysis, the “well-adjusted” class had the fewest participants in the present study, accounting for 20.3 % of the total. By contrast, the “highly difficult” class accounted for over 38 %. This may imply that many parents find raising their children difficult. The prevalence of emotional and behavioral problems among 5-year-olds inferred from this study was much higher than the 10 % predicted by Goodman [5]. In a previous study in China, LCA was conducted on five subscales of the SDQ, including the prosocial scale, with 4121 adolescents aged 11–18 years old who were categorized into three classes: “high difficulties” (34.8 %), “uncooperative” (35.3 %), and “well-adjusted” (29.9 %). In addition, the “uncooperative” class in the Chinese study showed no emotional or behavioral problems but low prosocial behavior, similar to our findings in the “asocial” class. Both Japanese and Chinese studies identified three similarly trending classes representing subtypes of children. This may reflect the children's characteristics and nurturing environment, as they are from the same Asian region. Although behavioral problems and hyperactivity are common during childhood, they do not necessarily continue at later ages [28]. Nevertheless, persistent extraversion externalizing symptoms may be a future risk factor for deviant behaviors, peer problems, and the development of emotional symptoms [28]; therefore, appropriate support from parents and teachers is needed for children with emerging problems.

We investigated risk factors for determining class assignments. In this analysis, we paid particular attention to whether sex, maternal postpartum depression, and mother-infant bonding were predictors of a specific class. Multinomial logistic regression results showed that the “asocial” and “highly difficult” classes had more males and higher MIBS scores at 10 months compared to the “well-adjusted” class. However, no factors differentiating these two classes were found. An SDQ survey in Japan [8] revealed that males have higher externalizing problem scores (conduct problems, hyperactivity) than females. However, the present study shows that males are more likely to be in the “asocial” (does not exhibit prosocial behaviors and has few problem behaviors) or “highly difficult” classes rather than the “well-adjusted” class. In a study conducted in China, no significant differences according to sex were found across classes [18]. Compared to the “well-adjusted” class in China, members of the other two classes were found to have significantly lower levels of maternal education. However, there were notable differences in the findings: in the Japanese study, class prediction was influenced by sex, while maternal education did not appear to significantly impact class assignment.

Mason [29] showed that maternal depression at two months postpartum affects the socio-emotional development of infants at six months, which is explained by the strength of mother-infant bonding at two months. In other words, one factor affecting the socio-emotional development of children is the maternal emotional bond with a child [17,29,30], and the present study showed similar results. Although this analysis has not revealed it, the possibility that a common third factor (e.g., genetics that is heritable, mode of delivery, breastfeeding, and involvement of paternal factors) may be causally influencing both maternal factors at 10 months and child behaviors at 5 years. Bonding insecurity may share a common neurobiological substrate with neurodevelopmental disorders [31]. A previous study showed that mothers who delivered via cesarean section (planned or unplanned) reported stronger parent-infant-bonding at 8 weeks and 14 months postpartum [32].Breastfeeding and active bonding with mothers significantly reduce the risk of children developing internalizing problems [33]. According to review studies, it has been suggested that the greater the father's involvement, the more likely it is that the child's problematic behavior will decrease [34,35]. The existence of which was revealed in this research, children who are more aggressive toward others and less prosocial exhibit maladaptive behaviors later in life [36]. Maintaining healthy social interactions is important for future success in work and life. In Japan, all families receive public health service visits within the first two months postpartum. The EPDS and MIBS questionnaires were used in most of them, and intensive care (such as hospital visits, the introduction of childcare services, and home visits by public health nurses) was provided to mothers with depressive tendencies or suspected mother-infant bonding disorders. Currently, in Japan, EPDS and MIBS are retested after two weeks only if scores are high (i.e., above 9). The results of the current study suggest that EPDS and MIBS at ten months predict the latent class derived from the child's strengths and difficulties at the age of five years. If necessary, the EPDS and MIBS questionnaires administered in municipalities can be used as maternal mental health screening instruments to determine whether to initiate public support not only at 0–1 month but also at 10 months postpartum.

The current study has several limitations. First, all questionnaires were self-reported and filled out by mothers. Thus, there was a lack of objective evaluation. Second, the questionnaire collection rate was 72.9 % (275 valid responses out of 379 births). It is possible that some of those who did not respond had more severe problems, which could have led to selection bias. Of the 379 participants, the 104 mothers who dropped out before their child was 5 years old and did not ultimately respond had lower levels of education than the 275 mothers who did respond. This may have affected the characteristics of the participants who did respond to the questionnaire. Higher rates of reported behavior problems in 5- to 6-year-old children were significantly associated with low parental education and occupation [37]. Therefore, it is possible that the fact that the participants who responded at the age of 5 had a significantly higher educational background than those who did not respond may have lowered the SDQ score. Third, the survey was conducted in suburban areas in Chiba and Saitama Prefectures, which border Tokyo and have populations of 6.2 and 7.2 million, respectively. Therefore, the sample cannot represent the entire population of children in Japan. Fourth, there is a lack of information about some factors. We could not compare the results of this study with those from China regarding marital status. Social support and satisfaction with support influence mother-infant bonding disorders and maternal depression [38]. As a form of such support, childcare services may also be useful as a public health intervention to mitigate the negative effects of maternal depression on children's internalizing problems [39]. However, the present study was not able to consider the presence or absence of social services, including support from public health nurses and the use of childcare services, to improve the mother's mental health and the parent-child relationship. In future studies, it will be important to collect these data and consider their impact.

Despite these limitations, our study demonstrates the utility of a person-centered approach in revealing the diversity of children's internalizing and externalizing symptoms and in highlighting the distinguishing characteristics of individuals within each subgroup. In conclusion, using the LCA, we identified three patterns of children's psychological strengths and difficulties. Being male and having insufficient maternal bonding at 10 months of age were predictors of “highly difficult” and “asocial” children at the age of five years. Parents and professionals can use these findings to develop adaptive prevention strategies and clinical interventions for children with problems.

## CRediT authorship contribution statement

**Tomoko Kawasaki:** Writing – original draft, Visualization, Project administration, Methodology, Formal analysis, Conceptualization. **Yoshikazu Noda:** Writing – review & editing, Validation, Methodology, Formal analysis, Conceptualization. **Yoshiyuki Hirano:** Writing – review & editing, Visualization, Supervision, Methodology, Formal analysis, Conceptualization. **Akiko Kawanami:** Writing – review & editing, Investigation. **Kenichi Sakurai:** Writing – review & editing, Investigation. **Chisato Mori:** Writing – review & editing, Supervision. **Eiji Shimizu:** Writing – review & editing, Supervision.

## Ethical approval statement

This study was approved by the Biomedical Research Ethics Committee of the Graduate School of Medicine, Chiba University (ID 451, approved on November 8, 2013, and updated to ID 1239, approved on March 29, 2023). All participants provided written informed consent.

## Data availability statement

The datasets generated and analyzed during the current study are available from the corresponding author upon reasonable request. However, the data are not publicly available due to the inclusion of information that could compromise the privacy of research participants.

## Funding

This work was supported by JSPS KAKENHI JP Grant Number JP16H01781 and Grants from the 10.13039/100019567Chiba Foundation for Health Promotion and Disease Prevention, Japan.

## Declaration of competing interest

The authors declare that they have no known competing financial interests or personal relationships that could have appeared to influence the work reported in this paper.

## Footnotes

Appendix A Supplementary data to this article can be found online at https://doi.org/10.1016/j.heliyon.2025.e41727.
