# Supplementary material for: The effects of mother-infant bonding on children's strengths and difficulties
Source: Heliyon. 2025 Jan 6;11(3):e41727. doi: 10.1016/j.heliyon.2025.e41727 (PMC11815701; doi:10.1016/j.heliyon.2025.e41727)
Supplement: Multimedia component 1 [file mmc1.pdf]

Appendix A: Strengths and Difficulties Questionnaire

For each item, please mark the box for Not True, Somewhat True or Certainly True. It would help us if you answered all items as best you can even if you are not absolutely certain or the item seems daft! Please give your answers on the basis of the child's behaviour over the last six months or this school year.

Child's Name .... *Male/Female*

Date of Birth..................

	Not True	Somewhat True	Certainly True
Considerate of other people's feelings	☐	☐	☐
Restless, overactive, cannot stay still for long	☐	☐	☐
Often complains of headaches, stomach-aches or sickness	☐	☐	☐
Shares readily with other children (treats, toys, pencils etc.)	☐	☐	☐
Often has temper tantrums or hot tempers	☐	☐	☐
Rather solitary, tends to play alone	☐	☐	☐
Generally obedient, usually does what adults request	☐	☐	☐
Many worries, often seems worried	☐	☐	☐
Helpful if someone is hurt, upset or feeling ill	☐	☐	☐
Constantly fidgeting or squirming	☐	☐	☐
Has at least one good friend	☐	☐	☐
Often fights with other children or bullies them	☐	☐	☐
Often unhappy, down-hearted or tearful	☐	☐	☐
Generally liked by other children	☐	☐	☐
Easily distracted, concentration wanders	☐	☐	☐
Nervous or clingy in new situations, easily loses confidence	☐	☐	☐
Kind to younger children	☐	☐	☐
Often lies or cheats	☐	☐	☐
Picked on or bullied by other children	☐	☐	☐
Often volunteers to help others (parents, teachers, other children)	☐	☐	☐
Thinks things out before acting	☐	☐	☐
Steals from home, school or elsewhere	☐	☐	☐
Gets on better with adults than with other children	☐	☐	☐
Many fears, easily scared	☐	☐	☐
Sees tasks through to the end, good attention span	☐	☐	☐

Signature .... Date ....

Parent/Teacher/Other (please specify:)

Thank you very much for your help

© Robert Goodman, 1977

Appendix B: Provisional Banding of SDQ Scores

These bands, which are not adjusted for age or gender, have been chosen so that roughly 80% *of children in the community are normal,* 10% *are borderline, and* 10% *are abnormal.*

	Normal	Borderline	Abnormal
Parent completed			
Total Difficulties Score	0–13	14–16	17–40
Emotional Symptoms Score	0–3	4	5–10
Conduct Problems Score	0–2	3	4–10
Hyperactivity Score	0–5	6	7–10
Peer Problems Score	0–2	3	4–10
Prosocial Behaviour Score	6–10	5	0– 4
Teacher completed			
Total Difficulties Score	0–11	12–15	16–40
Emotional Symptoms Score	0–4	5	6–10
Conduct Problems Score	0–2	3	4–10
Hyperactivity Score	0–5	6	7–10
Peer Problems Score	0–3	4	5–10
Prosocial Behaviour Score	6–10	5	0– 4
