# Supplementary material for: The effects of mother-infant bonding on children's strengths and difficulties
Source: Heliyon. 2025 Jan 6;11(3):e41727. doi: 10.1016/j.heliyon.2025.e41727 (PMC11815701; doi:10.1016/j.heliyon.2025.e41727)
Supplement: Multimedia component 2 [file mmc2.pdf]

Appendix

Mother-to-Infant Bonding Scale

I would like to know how you have been feeling about your baby lately. Listed below are some of the feelings mothers have about their babies. Please underline the answer which comes closest to how you usually feel about your baby, not just how you feel today. Please complete ALL items.

Here is an example,

I enjoy doing things for my baby

Not at all

<u>Slightly, some of the time</u>

Very much so, some of the time

Very much so, most of the time

This would mean: usually, "I slightly enjoy doing things for my baby, some of the time". Please complete the other questions the same way.

---

1) **I feel loving towards my baby**

Very much so, most of the time

Very much so, some of the time

Slightly, some of the time

Not at all

2) **I feel scared or panicky when I have to do something for my baby**

Not at all

Slightly, some of the time

Very much so, some of the time

Very much so, most of the time

3) **I feel resentful towards my baby**

Very much so, most of the time

Very much so, some of the time

Slightly, some of the time

Not at all

4) **I feel nothing for my baby**

Very much so, most of the time

Very much so, some of the time

Slightly, some of the time

Not at all

5) **I feel angry with my baby**

Very much so, most of the time

Very much so, some of the time

Slightly, some of the time

Not at all

6) **I enjoy doing things for my baby**

Not at all

Slightly, some of the time

Very much so, some of the time

Very much so, most of the time

7) **I wish my baby was different**

Very much so, most of the time

Very much so, some of the time

Slightly, some of the time

Not at all

8) **I feel protective towards my baby**

Not at all

Slightly, some of the time

Very much so, some of the time

Very much so, most of the time

9) **I wish I did not have my baby**

Very much so, most of the time

Very much so, some of the time

Slightly, some of the time

Not at all

10) **I feel close to my baby**

Not at all

Slightly, some of the time

Very much so, some of the time

Very much so, most of the time

Thank you for answering this questionnaire.
