# Supplementary material for: The effects of mother-infant bonding on children's strengths and difficulties
Source: Heliyon. 2025 Jan 6;11(3):e41727. doi: 10.1016/j.heliyon.2025.e41727 (PMC11815701; doi:10.1016/j.heliyon.2025.e41727)
Supplement: Multimedia component 3 [file mmc3.pdf]

Appendix
Edinburgh Postnatal Depression Scale (EPDS)

The Edinburgh Postnatal Depression Scale (EPDS) has been developed to assist primary care health professionals to detect mothers suffering from postnatal depression; a distressing disorder more prolonged than the 'blues' (which occur in the first week after delivery) but less severe than puerperal psychosis.

Previous studies have shown that postnatal depression affects at least 10% of women and that many depressed mothers remain untreated. These mothers may cope with their baby and with household tasks, but their enjoyment of life is seriously affected and it is possible that there are long-term effects on the family.

The EPDS was developed at health centres in Livingston and Edinburgh. It consists of ten short statements. The mother underlines which of the four possible responses is closest to how she has been feeling during the past week. Most mothers complete the scale without difficulty in less than 5 minutes.

The validation study showed that mothers who scored above a threshold 12/13 were likely to be suffering from a depressive illness of varying severity. Nevertheless the EPDS score should *not* override clinical judgement. A careful clinical assessment should be carried out to confirm the diagnosis. The scale indicates how the mother has felt *during the previous week*, and in doubtful cases it may be usefully repeated after 2 weeks. The scale will not detect mothers with anxiety neuroses, phobias or personality disorders.

Instructions for users

1. The mother is asked to underline the response which comes closest to how she has been feeling in the previous 7 days.
2. All ten items must be completed.
3. Care should be taken to avoid the possibility of the mother discussing her answers with others.
4. The mother should complete the scale herself, unless she has limited English or has difficulty with reading.
5. The EPDS may be used at 6–8 weeks to screen postnatal women. The child health clinic, postnatal check-up or a home visit may provide suitable opportunities for its completion.

EDINBURGH POSTNATAL DEPRESSION SCALE (EPDS)

J. L. Cox, J. M. Holden, R. Sagovsky
Department of Psychiatry, University of Edinburgh

Name:
Address:
Baby's age:

As you have recently had a baby, we would like to know how you are feeling. Please UNDERLINE the answer which comes closest to how you have felt IN THE PAST 7 DAYS, not just how you feel today.

Here is an example, already completed.
I have felt happy:
Yes, all the time
Yes, most of the time
No, not very often
No, not at all
This would mean: "I have felt happy most of the time" during the past week. Please complete the other questions in the same way.

In the past 7 days:

1. I have been able to laugh and see the funny side of things
   - As much as I always could
   - Not quite so much now
   - Definitely not so much now
   - Not at all
2. I have looked forward with enjoyment to things
   - As much as I ever did
   - Rather less than I used to
   - Definitely less than I used to
   - Hardly at all

* 3. I have blamed myself unnecessarily when things went wrong
   - Yes, most of the time
   - Yes, some of the time
   - Not very often
   - No, never

4. I have been anxious or worried for no good reason
   - No, not at all
   - Hardly ever
   - Yes, sometimes
   - Yes, very often

* 5. I have felt scared or panicky for no very good reason
   - Yes, quite a lot
   - Yes, sometimes
   - No, not much
   - No, not at all

* 6. Things have been getting on top of me
   - Yes, most of the time I haven't been able to cope at all
   - Yes, sometimes I haven't been coping as well as usual
   - No, most of the time I have coped quite well
   - No, I have been coping as well as ever

* 7. I have been so unhappy that I have had difficulty sleeping
   - Yes, most of the time
   - Yes, sometimes
   - Not very often
   - No, not at all

* 8. I have felt sad or miserable
   - Yes, most of the time
   - Yes, quite often
   - Not very often
   - No, not at all

* 9. I have been so unhappy that I have been crying
   - Yes, most of the time
   - Yes, quite often
   - Only occasionally
   - No, never

*10. The thought of harming myself has occurred to me
   - Yes, quite often
   - Sometimes
   - Hardly ever
   - Never

Response categories are scored 0, 1, 2, and 3 according to increased severity of the symptom.
Items marked with an asterisk are reverse scored (i.e. 3, 2, 1 and 0). The total score is calculated by adding together the scores for each of the ten items.
Users may reproduce the scale without further permission providing they respect copyright (which remains with the *British Journal of Psychiatry*) by quoting the names of the authors, the title and the source of the paper in all reproduced copies.

*J. L. Cox, MA, DM, FRCP(Edin), FRCPsych, *Professor of Psychiatry, Department of Postgraduate Medicine, University of Keele. Consultant Psychiatrist, City General Hospital, Stoke-on-Trent, formerly Senior Lecturer, Department of Psychiatry, University of Edinburgh;* J. M. Holden, BSc, SRN, HVCert, *Research Psychologist; R. Sagovsky, MB, ChB, MRCPsych, Research Psychiatrist, Department of Psychiatry, University of Edinburgh*

*Correspondence: *University of Keele, Thornburrow Drive, Hartshill, Stoke-on-Trent, Staffs SI7 7QB*

Downloaded from https://www.cambridge.org/core. 18 Jun 2021 at 06:18:19, subject to the Cambridge Core terms of use.
