# Supplementary material for: The effects of mother-infant bonding on children's strengths and difficulties
Source: Heliyon. 2025 Jan 6;11(3):e41727. doi: 10.1016/j.heliyon.2025.e41727 (PMC11815701; doi:10.1016/j.heliyon.2025.e41727)
Supplement: Multimedia component 4 [file mmc4.docx]

Supplementary Table 1. Sociodemographic variables depending on completed participants and those who dropped out by the age of 5

|  |  | All | Completed participants | Dropout until  5 years old | p-value | Effect size Cramer's V |
| --- | --- | --- | --- | --- | --- | --- |
|  |  |  |  |  |  |  |
|  |  | n=379 | n=275 | n=104 |  |  |
| Sex |  |  |  |  | n.s. |  |
| male (%) |  | 197 | 142 (51.6) | 55 (52.9) |  |  |
| female (%) |  | 182 | 133 (48.4) | 49 (47.1) |  |  |
| Mother's age, M (SD), (n=355) |  | 32.54 (4.7) | 32.62 (4.6) | 32.29 (4.2) | n.s. |  |
| First child (%) | Yes | 123 (44.7) | 123 (44.7) | n.a. |  |  |
|  | No | 152 (55.3) | 152 (55.3) | n.a. |  |  |
|  | Unclear | 104 | 0 | 104 |  |  |
| Mother's education, N (%) | Junior high school | 7 (2.0) | 4 (1.5) | 2 (4.0) | 0.032 | 0.16 |
|  | High school | 72 (22.0) | 53 (19.6) | 23 (30.3) |  |  |
|  | Junior college | 134 (38.7) | 114 (42.2) | 20 (26.3) |  |  |
|  | University/ graduate school | 129 (37.3) | 99 (36.7) | 30 (39.5) |  |  |
|  | Unclear | 33 (8.7) | 5(1.8) | 28 (26.9 ) |  |  |
| Household income, N (%) | < 2 million yen | 1 (0.3) | 1 (0.4) | 0 (0.0) | n.s. |  |
|  | 2-4 million yen | 59 (17.7) | 46 (17.7) | 13 (17.8) |  |  |
|  | 4-6 million yen | 105 (31.4) | 83 (31.9) | 22 (30.1) |  |  |
|  | 6-8 million yen | 88 (26.4) | 70 (26.9) | 18 (26.7) |  |  |
|  | 8-10 million yen | 40 (12.0) | 29 (11.2) | 11 (15.1) |  |  |
|  | 10-12 million yen | 24 (7.2) | 19 (7.3) | 5 (6.9) |  |  |
|  | 12-15 million yen | 10 (3.0) | 7 (2.7) | 3 (4.11) |  |  |
|  | 15-20 million yen | 3 (0.9) | 2 (0.8) | 1 (1.4) |  |  |
|  | > 20 million yen | 3 (0.9) | 3 (1.1) | 0 (0.0) |  |  |
|  | Unclear | 42 (11.1) | 15(5.5) | 31(29.8) |  |  |
| History of mother’s mental disorders, N (%) | Yes | 30 (9.2) | 20 (8.1) | 10 (13.0) | n.s. |  |
|  | No | 295 (90.8) | 227 (91.9) | 67 (87.0) |  |  |
|  | Unclear | 32 (8.4) | 28 (10.2) | 27 (26.0) |  |  |
| MIBS 10-month scores, M (SD) , (n=297） |  | 2.34 (2.68) | 2.42 (2.77) | 1.89 (2.07) | n.s. |  |
| EPDS 10-month scores, M (SD) , (n=301) |  | 4.00 (3.51) | 3.97 (3.45) | 4.18 (3.85) | n.s. |  |
| n.a.; This item was not collected at 5 years. | |  |  |  |  |  |
